# Candidate modifier genes for immune function in 22q11.2 deletion syndrome

**DOI:** 10.1002/mgg3.1057

**Published:** 2019-12-12

**Authors:** Catherina T. Pinnaro, Travis Henry, Heather J. Major, Mrutyunjaya Parida, Lucy E. DesJardin, John R. Manak, Benjamin W. Darbro

**Affiliations:** ^1^ Stead Family Department of Pediatrics Iowa City IA USA; ^2^ Iowa State Hygienic Laboratory Coralville IA USA; ^3^ Department of Biochemistry Iowa City IA USA; ^4^ Departments of Biology and Pediatrics University of Iowa Iowa City IA USA

**Keywords:** 22q11.2 deletion syndrome, genetic modifiers, immune dysregulation, retinoic acid

## Abstract

**Background:**

The 22q11.2 deletion syndrome (22q11.2DS) is the most common contiguous microdeletion affecting humans and exhibits extreme phenotypic heterogeneity. Patients can manifest any combination of comorbidities including congenital heart disease, hypoparathyroidism, cleft palate, kidney abnormalities, neurodevelopmental disorders, and immune dysfunction. Immunodeficiency is present in the majority of patients with 22q11.2DS and is the second leading cause of death in these patients. Knowing the genetic determinants of immune dysfunction will aid in prognostication and potentially novel treatments.

**Methods:**

We performed exome sequencing and gene‐based variant association analysis on 31 deeply phenotyped individuals with the canonical 3Mb 22q11.2 deletion to identify what genes outside the 22q11.2 locus may be modifying the immune dysregulated phenotype. Immunophenotyping was performed using preexisting medical data and a novel scoring system developed from numerous clinical laboratory values including immunoglobulin levels, lymphocyte transformation to antigens (LTA), lymphocyte transformation to mitogens (LTM), and peripheral blood flow cytometry. Immunophenotypic scoring was validated against newborn screening T‐cell receptor excision circle (TREC) results.

**Results:**

Rare DNA variants in transcriptional regulators involved in retinoic acid signaling (*NCOR2,* OMIM *600848 and *EP300*, OMIM *602700) were found to be associated with immunophenotype.

**Conclusion:**

The expression of *TBX1*, which seems to confer the major phenotypic features of 22q11.2DS, is regulated via retinoic acid signaling, and alterations in retinoic acid signaling during embryonic development can lead to phenocopies of 22q11.2DS. These observations support the hypothesis that genetic modifiers outside the microdeletion locus may influence the immune function in 22q11.2DS patients.

## Graphical abstract

We performed deep phenotyping for immune status on 31 individuals with the canonical 3Mb 22q11.2 deletion followed by whole exome sequencing. DNA variants in transcriptional regulators involved in retinoic acid signaling (NCOR2, EP300) were found to be associated with immune status.

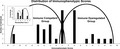

## 1 INTRODUCTION

22q11.2 deletion syndrome (22q11.2DS) is the most common microdeletion syndrome, affecting an estimated 1 in 6,000 to 1 in 3,000 live births (Botto et al., 2003; Devriendt, Fryns, Mortier, van Thienen, & Keymolen, 1998; Oskarsdottir, Vujic, & Fasth, 2004). Pathophysiology of the syndrome stems from dysembryogenesis of the third and fourth pharyngeal arches and leads to diverse clinical features including congenital heart defects, hypoparathyroidism, palatal abnormalities, and thymic hypoplasia or aplasia with resultant T‐cell dysfunction (Jackson et al., 2019; McDonald‐McGinn et al., 2015). Other clinical features of 22q11.2DS include renal anomalies, autoimmunity, developmental delays, and behavioral and psychiatric disorders (Van Batavia et al., 2019; Pereira & Marion, 2015; Tang et al., 2014).

There are 90 known or predicted genes present in the typical 3Mb 22q11.2 locus that are hemizygously deleted in 22q11.2DS (McDonald‐McGinn et al., 2015). *TBX1* is the most studied, and its deletion appears to be crucial to the development of the major components of the 22q11.2DS phenotype (Jerome & Papaioannou, 2001). However, despite the common genetic etiology of 22q11.2DS this condition also displays extreme phenotypic heterogeneity. The phenotype expressed is largely independent of deletion size and thus the constellation of other genes deleted (McDonald‐McGinn et al., 2015). Emerging work in this area has identified genetic drivers of kidney disease in the 22q11.2DS population, but the vast majority of phenotypic heterogeneity remains unexplained (Lopez‐Rivera et al., 2017). The ability to predict the degree and severity of a patient's clinical phenotype could allow for earlier detection of associated clinical features, which may improve patient‐specific outcomes as well as condition‐specific genetic counseling (Barry et al., 2017).

Some degree of immune deficiency affects up to 75% of patients with 22q11.2DS, and the dysregulation evolves with age (Crowley, Ruffner, McDonald McGinn, & Sullivan, 2018; McDonald‐McGinn et al., 2015). Infants typically have variable T‐cell lymphopenia that is related to thymic hypoplasia (Crowley et al., 2018). As such, newborn screening for severe combined immunodeficiency (SCID) has identified infants with 22q11.2DS due to T‐cell lymphopenia, and some recommend rapidly testing those individuals who screen positive for SCID but are then confirmed not to have SCID for 22q11.2DS even in the absence of typically associated features, particularly when B cells and NK cells are normal (Barry et al., 2017).

As mentioned above, the immune dysregulation tends to evolve with age, with the emergence of T‐cell dysfunction, and secondary humoral immune deficiency noted in older children and adults (Crowley et al., 2018). In addition, functional NK cell deficiency has also been demonstrated in patients with 22q11.2DS, which may be attributed to *CRKL* haploinsufficiency occurring in the majority of these patients (Zheng et al., 2015). Additionally, autoimmunity and atopy are prevalent, and studies have demonstrated an evolution of early Th1 production skewing toward a Th2 cytokine profile in adults, consistent with an atopic phenotype (Zemble et al., 2010).

Recent studies have identified modifiers outside of the 22q11.2 region associated with congenital heart disease phenotype and variations in palatal phenotype (Driscoll et al., 2006; Guo et al., 2015, 2017). We hypothesized that genetic modifiers (also referred to as an individual's genetic background) contribute to variable expressivity and penetrance in 22q11.2 DS. The purpose of this study was to identify candidate genetic variants that influence immunophenotype in 22q11.2DS patients.

## 2 MATERIALS AND METHODS

### 2.1 Patients, phenotyping, and score validation

**Figure 1:**
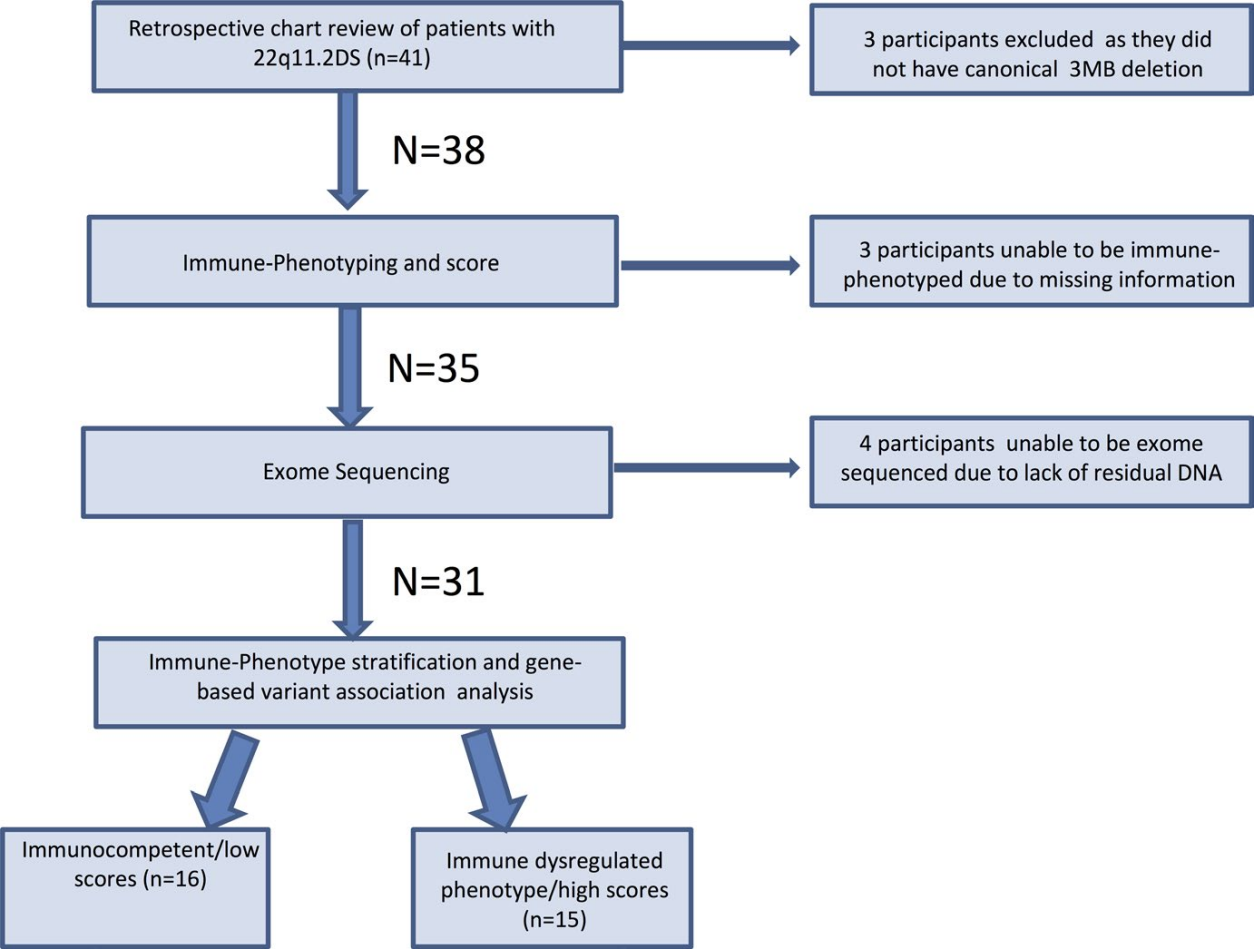
Flow diagram of participant ascertainment

Following the approval by the Iowa Department of Public Health and our Institutional Review Board, focused newborn screen information and medical records were reviewed for 41 patients with 22q11.2 deletions. These patients were identified through the University of Iowa Division of Medical Genetics, the University of Iowa Immune Disorders clinic, and the Shivanand R. Patil Cytogenetics and Molecular Laboratory. Thirty‐eight patients had the canonical 3Mb 22q11.2 deletion (breakpoints A‐D); 35 individuals had enough medical information to perform deep phenotyping. Of these 35 individuals, 4 did not have enough residual DNA following clinical chromosomal microarray testing to undergo exome sequencing. A total of 31 individuals were thus included in this study (Figure 1).

Patients were phenotyped for traditional 22q11.2DS abnormalities and specifically for immune function. To quantify the degree of immune dysregulation in our patients, we created an immunophenotypic scoring system utilizing numerous laboratory results often obtained following a diagnosis of 22q11.2DS. These laboratory values included immunoglobulin levels, lymphocyte transformation to antigens (LTA) and mitogens (LTM), and peripheral blood flow cytometry values. Patient laboratory values were compared to age‐specific normative values (Shearer et al., 2003; Soldin & Wong 2005). Tests involving the use of monoclonal and/or polyclonal antibodies or recombinant cytokines employed normative values determined by the VA Diagnostic Immunology Laboratory of the Iowa City Veterans Administration Medical Center. Patients were followed for a mean of 20.3 months. There was no difference in follow‐up duration when comparing the immunocompetent to the immune dysregulated groups (23.3 vs. 17.9 months, *p* = .45).

For each individual laboratory value considered, one point was assigned (+1) for each documented abnormal result (i.e., if the patient had a lab value lying outside of the age‐specific normative range for the laboratory metric of interest) and one point was subtracted (−1) for each normal result. For example, if at the time of chart review, a patient had three normal IgG laboratory value results and one elevated result the total score would be −2. This scoring was performed for a total of 37 individual laboratory tests. If two or more laboratory tests had identical scores across all patients, one of the tests was removed from further consideration randomly. If two or more laboratory tests had a Pearson's correlation coefficient of greater than 90% across all patients the test with the least number of patient values was removed from further consideration. In the event a patient did not have a specific laboratory test performed, the median value of all other patient scores was used, however, if a patient was missing over half of the laboratory tests under consideration they were removed from further analysis and likewise, if a specific laboratory test was not performed on at least 75% of the patients then that laboratory test was removed from further consideration. These steps resulted in the removal of 12 laboratory tests from further consideration.

After transformation of the normal/abnormal laboratory values into integers, we subsequently performed pairwise Pearson correlation testing of all individual laboratory tests against T‐cell receptor excision circle (TREC) values reported from the newborn screen. TRECs represent circularized, excised pieces of DNA that are generated during sequential rearrangement of the T‐cell receptor gene (Serana et al., 2013). The TREC assay was performed by the State of Iowa Hygienic Laboratory as part of the routine newborn screening process. The TREC assay methodology has been previously described (Mirza, Phillips, Henry, & Fasano, 2014). T‐cell lymphopenia correlates with low or absent TRECs and has successfully identified known SCID and other selected T‐cell deficient patients (Gul et al., 2015; Mirza et al., 2014). Thus, TREC should be a good validity indicator for our immunophenotypic scoring system, since 22q11.2 deletion syndrome is associated with embryological disruption of the third and fourth pharyngeal arches, leading to hypoplasia and rarely aplasia of the thymus and thus varying degrees of T‐cell deficiency (Maggadottir & Sullivan, 2013; McDonald‐McGinn et al., 2015). Additionally, infants with 22q11.2DS have a propensity to screen positive for SCID on TREC‐based newborn screening, so we hypothesized that the most representative set of values to quantify immune dysregulation would highly correlate with TREC values (Barry et al., 2017).

In addition to individual laboratory test pairwise correlation with TREC values, we also performed combinatoric calculations to determine if a combination of laboratory value scores would provide a better correlation with TREC than any individual laboratory test. Custom Python scripts utilizing the itertools, scipy, and pandas libraries were used to perform the combinatorics analysis. When considering a combination of laboratory test scores, both additive and subtractive values of the laboratory tests were considered. For example, the combination metric might include the addition of several laboratory test values to one another as well as the subtraction of others. Extensive testing showed a plateau in correlation coefficient once nine laboratory test metrics were included in the combination score. We subsequently chose the combination of nine laboratory test metrics with the highest correlation coefficient with the TREC value. The combination of laboratory values with the second highest correlation coefficient was also retained as it was used to stratify a subset of patients that obtained the same immunophenotypic score using the best combination metric.

### 2.2 Exome Sequencing and Association Analysis

Exome sequencing was performed as previously described (Brophy et al., 2017). Residual DNA samples were obtained from the Shivanand R. Patil Cytogenetics and Molecular Laboratory following clinical diagnostic chromosomal microarray testing which identified the 22q11.2 microdeletion. Samples were sequenced at the University of Iowa Institute of Human Genetics, Genomics Division, using the Agilent SureSelect Human All Exon Version 5 enrichment kit (Agilent Technologies, Santa Clara, CA) and an Illumina HiSeq2500 (Illumina, San Diego, CA). Data analysis was performed as previously described (Brophy et al., 2017) to generate variant call files for each individual. Variants were quality controlled and filtered by population allele frequency prior to further analysis. The allele frequency threshold used was 5% as indicated in either the 1,000 Genomes Project (1 kg) or the gNOMAD database (Auton et al., 2015; Lek et al., 2016). Filtered variants were then used in a gene‐based variant association analysis using VAAST 2.0 (Hu et al., 2013). VAAST 2.0 uses an aggregative variant association test that combines amino acid substitution, allele frequencies, and phylogenetic conservation to rank genes based on the presence of presumably functional variations differing in two cohorts. Following separation into two immunophenotypic groups, the VAAST 2.0 algorithm was run assuming dominant inheritance, incomplete penetrance, and locus heterogeneity. The results were then sorted by the VAAST 2.0 probability score. Variants identified in high ranking genes were subjected to in silico pathogenicity prediction algorithms including SIFT and PolyPhen‐2 (Adzhubei, Jordan, & Sunyaev, 2013; Ng & Henikoff, 2003).

## 3 RESULTS

### 3.1 Phenotyping

A total of 38 individuals with the canonical 3Mb deletion were assessed for traditional 22q11.2 phenotypes. Our population of patients with 22q11.2 DS exhibited these phenotypes consistent with previously published literature (Table 1). Immunophenotyping was then performed on 34 patients. Of the 37 relevant laboratory tests reviewed for immune status, 12 tests were removed from further analysis as they had identical values across all patients to another test or were highly correlated with another test (Table 2). Given that many immunoglobulins, especially IgG, within a newborn are derived from the mother we decided to exclude these laboratory values from further analysis. The remaining 23 laboratory test values were investigated for their correlation with 23 patient's TREC values. Five laboratory test values were individually correlated with TREC levels at a statistically significant level (*p < .05*). These tests included the relative levels of CD19 + and CD3 + cells, the absolute levels of CD3+/CD8 + and CD3‐/CD56 + cells, and the CD4:CD8 ratio.

**Table 1:** Clinical characteristics of 22q11.2DS patients

Characteristic	Total number	Observed percentage	Expected percentage(McDonald‐McGinn et al., 2015)
Hypocalcemia	19	51% (19/37)	~50%
Congenital heart defect		50% (19/38)	~75%
Tetralogy of Fallot	6		
Interrupted Aortic Arch type B	5		
Truncus arteriosus	0		
Isolated VSD	4		
Other	4		
Palatal defects	15	45% (15/33)	~75%
VPI	12		
Submucous cleft	9		
Bifid/absent uvula	2		
Overt cleft	2		
Renal anomalies	7	24% (7/29)	~30%

Note

The denominator varies, as not all eligible patients had all data. The numerator for palatal defects is not summative, as several patients had multiple palate findings. The denominator for palatal defects is significantly lower than the number of patients included in our study, as many patients had not seen speech or ENT and therefore did not have these findings commented on in the medical record. The denominator for renal anomalies is lower than the number of patients in our study, as only 29 patients had screening renal ultrasounds performed at the time of this study.

**Table 2:** Clinical laboratory tests used to perform immunophenotyping

Immunoglobulins: IgG, IgA, and IgM
Lymphocyte Transformation to Antigens (LTA): *Alone, tetanus, candida, alloantigen*
Lymphocyte Transformation to Antigens (LTM): Alone, **conA**, **PHA**, PWM, IL2, **anti‐CD3**
Peripheral Blood Flow Cytometry: Absolute lymphocyte count, absolute levels of CD3+, CD19+, CD3+/CD4+, CD3+/CD8+, CD3+/CD4‐/CD8‐, *CD3+/CD56+*, CD3‐/CD56+, *CD4+/CD25+, CD8+/CD25+, CD3+/CD25+*, relative levels of CD3+, **CD19+, CD3+/CD4+, CD3+/CD8+,** *CD3+/CD4‐/CD8‐*, **CD3+/CD56+,** CD3‐/CD56+, **CD4+/CD25+,** CD8+/CD25+, *CD3+/CD25+, CD25+, CD14 + *cells, and the **CD4+/CD8 + cell ratio.**

Note

Italicized entries are those laboratory tests that were removed from further consideration because either less than 75% of 22q11.2DS patients had those tests performed or they were identical or highly correlated with another laboratory test result across all patients. Entries in bold are those used for the final combination immune dysregulation phenotype score.

Abbreviations: conA, concanavalin A; PHA, phytohemagglutinin; PWM, pokeweed mitogen.

**Figure 2:**
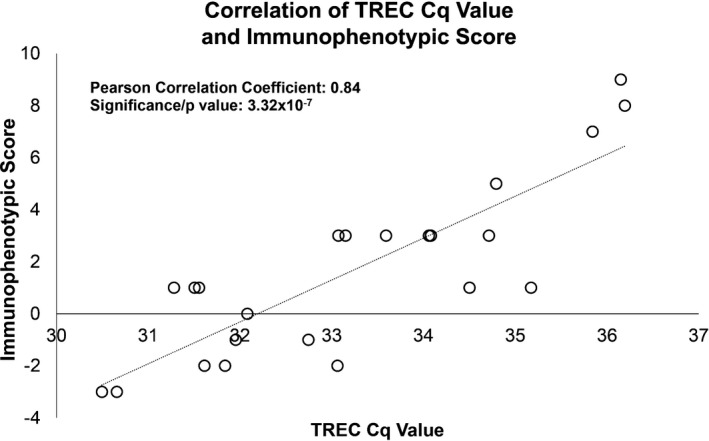
Correlation of the final immunophenotypic score versus the TREC Cq value obtained from newborn screening blood spot card. R^2^ = 0.84, *p* = 3.32 × 10^–7^

**Figure 3:**
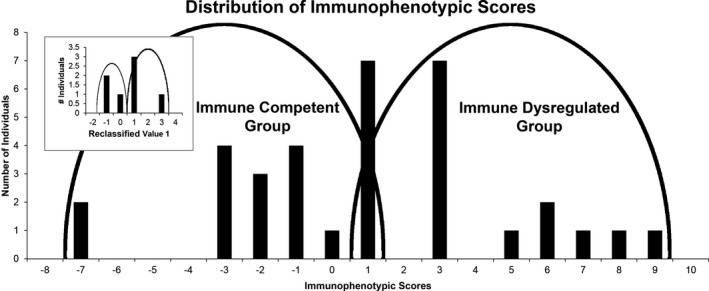
Distribution of immunophenotypic scores. Histogram represents the stratification of participants into the immunocompetent phenotype (scores ranging from −7 to + 1) and an immune dysregulated phenotype group (scores ranging from +1 to +9). Seven patients obtained an immunophenotyping score of 1 and required additional classification into either an immunocompetent or immune dysregulated group. These participants were substratified using the second best combination metric obtained from the combinatoric calculations

Although each of these metrics was individually significantly associated with TREC scores, we believed a combination of these and/or other metrics might yield a better correlation and thus more accurately reflect what TREC values may have been in those patients who were born before the testing became part of the Iowa Newborn Screen. Using a combinatorics framework we found a combination of nine individual laboratory values correlated with TREC values with a Pearson correlation coefficient of 0.84 and significance value of approximately 3.32 × 10^−7^ (Figure 2). The final combination metric included the sum of the lymphocyte transformation mitogen (LTM) tests anti‐CD3 and concanavalin A, the relative levels of CD3+/56+, CD3+/CD4+, and CD19 + cells, and the CD4:CD8 ratio as well as the subtraction of the values from the LTM–phytohemagglutinin (PHA) and the relative levels of CD3+/CD8 + and CD4+/CD25 + cells. Despite not reaching individual statistical significance, both LTM–PHA and CD4+/CD25 + were negatively correlated with TREC values. Using this combination score metric, patients were subsequently stratified into an immunocompetent phenotype group (scores ranging from −7 to +1) and an immune dysregulated phenotype group (scores ranging from +1 to +9). Seven patients obtained an immunophenotypic score of 1 and required additional classification into either an immunocompetent or immune dysregulated group. For this task we employed the second best combination metric obtained from the combinatoric calculations (Figure S1). Ultimately, 17 patients were included in the immune dysregulated group and 17 in the immunocompetent group (Figure 3).

Of note, our scoring system did not produce a clear division of 75% of patients with immune dysregulation. This may be because those with the most severe phenotypes were unable to be included in the study. Three of the five subjects who were excluded from analysis due to lack of available immunophenotypic information died before immunologic workup could be initiated and may represent the most extreme phenotype in our cohort.

### 3.2 Sequencing and association analysis

After dividing our cohort into two groups representing two different immunophenotypes, all individuals that had residual DNA remaining after clinical chromosomal microarray analysis had exome sequencing performed. Two individuals from the immune dysregulated group and one from the immunocompetent group were unable to be sequenced due to a lack of DNA. In total, 15 immune dysregulated individuals and 16 immunocompetent individuals with 22q11.2DS underwent exome sequencing (Table S1).

Since genetic modifier variants are not strictly required to be rare, we used a permissive minor allele frequency for variant filtering (5% in 1KG and gNOMAD). We used VAAST 2.0 to perform a gene‐based, aggregative variant association test looking for genes and variants that could increase the risk of immune dysregulation or protect against its development. We found 31 statistically significant genes that may play a protective role against immune dysregulation in 22q11.2DS and 50 statistically significant genes that may increase one's risk (Tables S2 and S3). In total, we identified >80 statistically significant candidate modifier genes of immune function between the two groups. Ingenuity pathway analysis (Qiagen Inc., Hilden, Germany) was performed to find gene sets enriched for by our candidate genes and highlighted two transcriptional regulators involved in retinoic acid signaling (*NCOR2,* OMIM *600,848 and *EP300*, OMIM *602700).

There were six unique *NCOR2* missense variations in seven immunocompetent individuals and six unique *EP300* missense variations in immune dysregulated individuals (Table 3). All variants identified in these two genes were missense mutations, and despite analyzing variants that had population frequencies as high as 5% all of those identified in these transcriptional regulators were rare (median minor allele frequency of 0.1%). Despite the rarity of these variations, the majority were predicted by both SIFT and PolyPhen2 to be tolerated/benign. Also notable is that all but one of the *NCOR2* and *EP300* mutations consistently missed functional domains. This is consistent with results from a prior genetic modifier study of cardiac phenotype in 22q11.2DS, where 18 of 21 variants in their 3 candidate modifiers missed functional domains (Guo et al., 2015). The one *NCOR2* variant that did occur within a functional domain was found in an individual within the immune dysregulated group suggesting it may have a different function than the rest of the variants discovered in this gene.

**Table 3:** Transcriptional regulation genes and DNA variants found in 22q11.2DS patients in either the immune dysregulated (high) or immunocompetent (low) score group

Gene Name	Variations	Individuals with Variant (and Group)	Frequency in gnomAD	SIFT	PolyPhen2
*NCOR2*	c.7166A>G, p.His2389Arg	1 (low)	3.28–05	Tolerated	Benign
	c.6569C>T, p.Pro2190Leu	1 (low)	0.0002	Damaging	Possibly Damaging
	c.5290G>A, p.Ala1764Thr	1 (low)	0.0010	Tolerated	Benign
	c.4552T>A, p.Ser1518Thr	1 (low)	0.0023	Tolerated	Benign
	c.2875A>C, p.Lys959Gln	1 (low)	7.04E−06	Damaging	Probably Damaging
	c.2545G>A, p.Glu849Lys	2 (low)	0.017	Tolerated	Benign
	c.1915C>T, p.Arg639Trp	1 (high)	8.14E−06	Damaging	Probably Damaging
*EP300*	c.6668A>C, p.Gln2223Pro	2 (high)	0.0283	Tolerated	Benign
	c.865A>G, p.Met289Val	1 (high)	0.0026	Tolerated	Benign
	c.3811G>A, p.Val1271Ile	1 (high)	0.0004	Tolerated	Benign
	c.7225A>C, p.Ser2409Arg	1 (high)	–	Damaging	Possibly Damaging
	c.2091T>G, p.Ser697Arg	1 (high)	0.0041	Damaging	Possibly Damaging
	c.6950G>A, p.Arg2317Gln	1 (high)	9.70E−05	Tolerated	Benign

Note

*NCOR2* NC_000012.12, *EP300* NC_000022.11.

## 4 DISCUSSION

The majority of patients with 22q11.2DS have some degree of thymic hypoplasia, and about 75% of children experience immunodeficiency (McDonald‐McGinn et al., 2015; Pereira & Marion, 2015). It is important to understand what distinguishes the other 25% of children with 22q11.2DS, as it may yield important genetic information with predictive significance that could allow for changes in care ultimately improving clinical outcomes (Barry et al., 2017). It may also provide information to improve genetic counseling, which may become more important due to increasing numbers of adults with 22q11.2DS having children (McDonald‐McGinn & Sullivan, 2011). If protective genetic variants are identified, those pathways could be targeted for the development of therapeutics to prevent some of the complications of 22q11.2DS before they manifest (Harper, Nayee, & Topol, 2015).

About 85% of patients with 22q11.2DS have a similar 3Mb deletion and another 5%–10% have the same proximal breakpoint but a nested distal breakpoint resulting in a 1.5Mb deletion (Edelmann, Pandita, & Morrow, 1999). There is variable expressivity and penetrance even among patients with identical deletions, and the majority of studies have not demonstrated a correlation between size and location of deletions and disease phenotype (Goodship, Cross, Scambler, & Burn, 1995; Kerstjens‐Frederikse et al., 1999; Michaelovsky et al., 2012). The *TBX1* gene does, however, seem to confer the major phenotypic features of the disorder. The development of *Tbx1* haploinsufficient mice produced similar cardiac, thymic, and parathyroid phenotypes to that of patients with 22q11.2DS, although none of the mice in *Tbx1* studies expressed the full spectrum of phenotypes observed in 22q11.2DS patients. These studies did demonstrate abnormal development of the pharyngeal arches and pouches, which is felt to be the pathogenesis for the thymic, parathyroid, palatal, and cardiac features of 22q11.2 deletion syndrome (Jerome & Papaioannou, 2001; Lindsay et al., 2001; Merscher et al., 2001; Pereira & Marion, 2015).

The penetrance of cardiovascular and other defects in 22q11.2DS is greatly affected by genetic background in mice, and individuals with 22q11.2DS and a congenital heart defect are more likely to have an unaffected relative with isolated congenital heart disease than individuals with 22q11.2DS with normal intracardiac and aortic arch anatomy (Swaby et al., 2011; Taddei, Morishima, Huynh, & Lindsay, 2001). This led us to hypothesize that other genes outside of the known disease‐associated area may be modifying the expression of the 22q11.2DS phenotype. We chose to look for both protective and risk alleles given that almost all patients with 22q11.2DS are hemizygous for *TBX1*, yet the degree to which these patients express the associated pharyngeal arch phenotype varies.

*TBX1* expression is regulated through the retinoic acid pathway, and alterations in retinoic acid during development can lead to phenocopies of 22q11.2DS (Lammer et al., 1985; Roberts, Ivins, James, & Scambler, 2005; Yu, Gonzalez, Martinez, Diez‐Pardo, & Tovar, 2003). Interestingly, the number one ranked gene in our VAAST analysis was *EP300*. Mutations in *EP300* were present significantly more often in the immune dysregulated group. When retinoic acid is present, transcriptional coactivator p300, via its cysteine/histidine motif, interacts with retinoid‐X receptor and forms a coactivator complex that increases subsequent gene transcription via histone acetyltransferase activity (Chen & Li, 2011; Kashyap & Gudas, 2010). Thus, mutations in this gene could also alter the transcription of important downstream products of retinoic acid signaling that are crucial in early embryogenesis. However, the included pathogenicity predictors produced varied results and several of the mutations were predicted to be tolerated. This will require further exploration.

Interestingly, another modulator of retinoic acid signaling was prominent in our results. Mutations in *NCOR2* were present significantly more often in the immunocompetent group. The protein product of *NCOR2* forms a corepressor complex with histone deacetylase activity that represses the expression of retinoic acid‐inducible genes (Minucci & Pelicci, 1999). Retinaldehyde dehydrogenase 2 (*Raldh2*) is the main generator of retinoic acid during vertebrate embryogenesis (Duester, 2008). Rald2^−/−^ mutants demonstrate alterations in levels of FGF8 and are phenocopies of 22q11.2DS (Kumar, Cunningham, & Duester, 2016; Niederreither et al., 2003). A recent study demonstrated that NCOR1 and NCOR2 redundantly mediate the ability of retinoic acid to repress FGF8 (Kumar et al., 2016). Thus, the mutations in *NCOR2* may provide important dose‐modulating upstream effects on retinoic acid, thus having downstream dose effects on FGF8 and TBX1. This could explain a potential role for *NCOR2* in modifying the phenotypic severity of 22q11.2DS. As seen with *EP300,* pathogenicity predictions did not display universal intolerance.

In addition to playing a crucial role in embryogenesis, which could impact several of the 22q.112DS phenotypes, retinoic acid via vitamin A is known to play an important role in childhood immunologic health. Vitamin A deficiency is associated with impaired vaccine responsiveness and increased morbidity and mortality to childhood diseases (Oliveira, Teixeira, & Sato, 2018; Semba, 1999). Additionally, retinoic acid has extensive action at a cellular level in both the innate and adaptive immune systems. For example, retinoic acid signaling through the retinoic acid alpha receptor induces the production of proinflammatory cytokines by dendritic cells thus triggering the differentiation of effector T cells (Hall et al., 2011). Retinoic acid signaling in B cells is necessary for B cell differentiation and IgA production from mouse intestinal cells (Pantazi et al., 2015). Thus, there is biologic plausibility for augmented retinoic acid signaling in modulating immunophenotype in 22q11.2DS.

The relatively small number of patients is a weakness of the study, and next steps include replicating the study with a larger sample size to determine if we again identify the same candidate modifier genes. Functional studies will be necessary to validate the results if replicated. There were several members of our cohort who inherited the deletion from a parent. However, the retrospective nature of data collection and use of newborn blood spot cards for DNA extraction excluded the parents from participation in this study. Further evaluating these individuals in a trio‐based context may also provide a pathway to evaluating function.

Another interesting finding of this study was the phenotypic scoring system itself. We were not surprised to note that the majority of markers significantly associated with TREC were T cell in nature. However, NK cells were also included in the most highly correlated score. Given the spectrum of immunologic abnormalities that evolves with age along with evidence that *CRKL* haploinsufficiency is associated with NK cell dysfunction, this could also indicate a potential mechanism for future study (Crowley et al., 2018; Zheng et al., 2015).

## Conflict of Interest

The authors have no conflict of interest.

## AUTHOR CONTRIBUTION

CTP contributed to creation of the scoring system and analysis, performed the immunophenotyping, and wrote the manuscript. TH and LED obtained and analyzed the TREC data from the newborn screen. HJM identified patients from CMA data and extracted and prepared DNA for exome sequencing. MP performed the combinatorics analysis. BWD conceived the idea for the project, obtained project funding, created the exome sequencing pipeline, performed the genetic analyses, and edited the manuscript.

## Data Availability Statement

The variant call files that support the findings of this study are available from the corresponding author upon reasonable request, however, the raw exome data are not available.
