# Supplementary figures and images for: Candidate modifier genes for immune function in 22q11.2 deletion syndrome
Source: Mol Genet Genomic Med. 2019 Dec 12;8(1):e1057. doi: 10.1002/mgg3.1057 (PMC6978229; doi:10.1002/mgg3.1057)

# Correlation of TREC Cq and Second Best Immunophenotypic Score

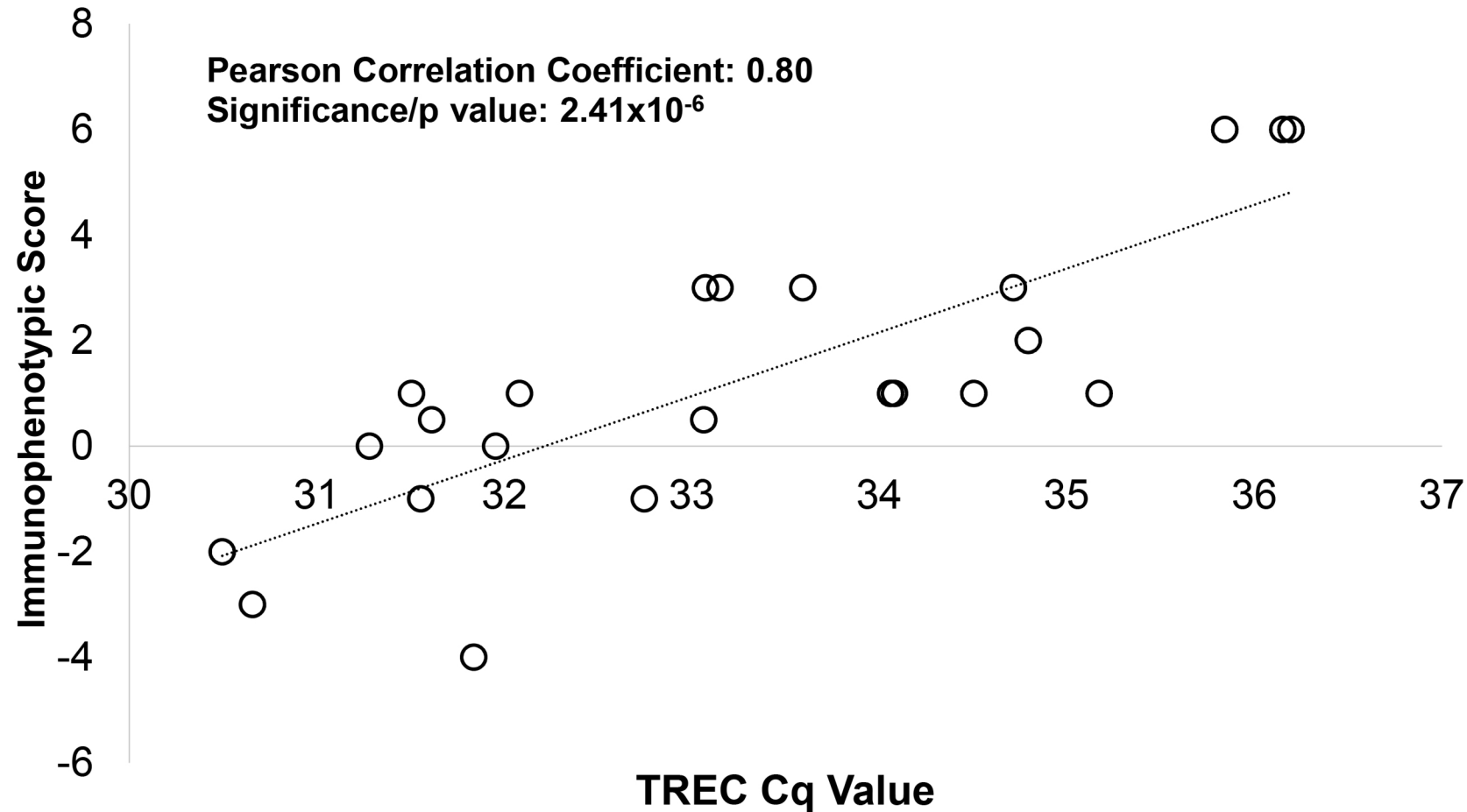

Supplement: Supplementary file 1 [file MGG3-8-e1057-s001.pdf]
