# Supplementary material for: Candidate modifier genes for immune function in 22q11.2 deletion syndrome
Source: Mol Genet Genomic Med. 2019 Dec 12;8(1):e1057. doi: 10.1002/mgg3.1057 (PMC6978229; doi:10.1002/mgg3.1057)
Supplement: Supplementary file 2 [file MGG3-8-e1057-s002.docx]

| PatientID | Notes | Cq TREC | Immunophenotypic Score Category | IgM qual | IgA qual | LTA-alone | LTA-tetanus | LTA- candida | LTA- alloantigen | LTM-alone | LTM- conA | LTM- PHA | LTM-PWM | LTM-IL2 | LTM- antiCD3 | Abs lymph count (qual) | Rel % CD19 | Abs CD19 | Rel % CD3 | Abs CD3 | Rel % CD3+/4+ | Abs CD3+/4+ | Rel % CD3+/CD8+ | Abs CD3+/8+ | CD4:CD8 Ratio | Rel % CD3+/4-/8- | Rel % CD3+/56+ | Abs CD3+/56+ | Rel % CD3-/56+ (Rel % CD56) | Abs CD3-/56+ (Abs CD56) | Rel % CD4+/25+ | Rel % CD8+/25+ | Abs CD8+/25+ | Rel % CD3+/25+ | CD14 | CD25 | Second Best Combination Metric | Best Combination Metric | Immunophenotypic Score Category |
| --- | --- | --- | --- | --- | --- | --- | --- | --- | --- | --- | --- | --- | --- | --- | --- | --- | --- | --- | --- | --- | --- | --- | --- | --- | --- | --- | --- | --- | --- | --- | --- | --- | --- | --- | --- | --- | --- | --- | --- |
| 4 |  | 31.506 | High | -1.5 | -1 | -1 | -1 | -1 | -1 | -1 | -1 | -1 | -1 | -1 | -1 | -1 | -1 | -1 | 1 | 1 | 1 | 1 | 1 | 1 | 1 | -1 | 1 | 1 | 1 | -1 | -1 | 1 | 1 | -1 | -1 | -1 | 1 | 1 | High |
| 12 |  | 33.073 | High | 1 | -1 | -1 | -1 | -1 | -1 | -1 | -1 | -1 | -1 | -1 | -1 | 1 | 1 | 1 | 1 | 1 | 1 | 1 | -1 | 1 | 1 | -1 | -1 | 1 | -1 | -1 | -1 | -1 | 1 | -1 | 1 | -1 | 3 | 3 | High |
| 13 |  | 33.15 | High | -2 | 0 | 0 | 0 | -2 | -2 | -1 | -1 | -1 | -1 | -1 | -1 | 1 | 3 | -1 | 3 | 3 | 3 | 3 | -1 | 1 | -3 | -3 | -3 | 1 | -3 | -3 | -3 | -3 | 1 | -1 | 1 | -1 | 3 | 3 | High |
| 14 |  | 33.592 | High | -5 | -1 | 0 | -2 | 2 | 0 | -1 | -1 | -1 | -1 | -1 | -1 | -1 | 1 | -1 | -1 | -3 | 1 | -3 | -3 | -3 | -3 | -1 | 1 | 2 | -3 | -3 | -1 | 1 | 2 | 0 | 2 | 0 | 3 | 3 | High |
| 15 |  | 34.06 | High | -2 | -1 | -1 | -1 | 1 | 1 | -1 | -1 | -1 | -1 | -1 | -1 | -3 | 1 | -1 | 3 | 1 | 3 | 1 | 3 | 1 | 3 | -3 | -3 | -2 | -1 | -1 | -3 | -3 | 1 | -1 | 1 | -1 | 1 | 3 | High |
| 16 |  | 34.081 | High | -1 | -1 | -1 | -1 | -1 | -1 | 1 | 1 | 1 | 1 | -1 | 1 | -1 | -1 | -1 | -1 | -1 | 1 | -1 | 1 | -1 | 1 | -1 | 1 | 1 | -1 | -1 | -1 | 1 | 1 | -1 | 1 | -1 | 1 | 3 | High |
| 17 |  | 34.503 | High | -1 | -1 | -1 | -1 | -1 | -1 | -1 | 1 | -1 | -1 | -1 | -1 | -1 | 1 | 1 | 1 | -1 | -1 | -1 | 1 | -1 | 1 | -1 | -1 | 1 | -1 | -1 | -1 | -1 | 1 | -1 | 1 | -1 | 1 | 1 | High |
| 18 |  | 34.714 | High | 1 | -1 | -1 | -1 | -1 | -1 | -1 | -1 | -1 | -1 | -1 | -1 | 1 | 1 | -1 | 1 | 1 | 1 | 1 | 1 | 1 | 1 | 1 | 1 | 1 | 1 | -1 | -1 | 1 | 1 | -1 | 1 | -1 | 3 | 3 | High |
| 19 |  | 34.793 | High | -2 | -2 | -1 | -1 | -1 | -1 | -1 | -1 | -1 | -1 | -1 | -1 | 0 | 2 | -2 | 2 | 0 | 0 | 0 | 2 | 0 | 2 | -2 | 2 | 2 | -2 | -2 | -2 | -2 | -2 | -2 | 2 | -2 | 2 | 5 | High |
| 20 |  | 35.172 | High | -3 | -3 | -1 | -1 | -1 | -1 | -1 | -1 | -1 | -1 | -1 | -1 | -1 | -1 | -1 | 1 | 1 | 1 | 1 | -1 | 1 | -3 | -3 | 1 | 1 | 1 | -1 | -3 | 1 | 2 | -2 | 2 | -2 | 1 | 1 | High |
| 22 |  | 36.151 | High | -5 | -3 | 0 | 0 | 2 | 0 | -1 | -1 | -1 | -1 | -1 | -1 | -2 | 8 | -2 | 4 | -2 | 0 | -6 | 2 | 2 | 0 | -6 | 0 | 3 | -6 | -8 | -4 | 0 | 3 | -1 | 3 | -1 | 6 | 9 | High |
| 31 |  |  | High | -1 | -1 | -1 | -1 | -1 | -1 | -1 | -1 | -1 | -1 | -1 | -1 | -1 | 1 | -1 | 1 | -1 | 1 | -1 | -1 | -1 | -1 | -1 | -1 | -1 | -1 | -1 | -1 | 1 | 1 | -1 | 1 | -1 | 3 | 1 | High |
| 32 |  |  | High | -2 | 0 | -1 | -1 | -1 | -1 | -1 | -1 | -1 | -1 | -1 | -1 | 0 | 2 | -2 | 0 | 2 | 2 | -2 | 0 | 2 | -2 | -2 | 2 | 2 | 0 | -2 | 0 | 2 | 2 | 0 | 0 | -2 | 2 | 3 | High |
| 33 |  |  | High | -1 | -3 | -1 | -1 | -1 | -1 | -2 | -2 | -2 | 0 | 0 | -2 | -4 | 3 | -1 | 4 | -2 | 4 | -2 | 4 | 0 | -1 | -3 | 3 | 3 | -3 | -3 | -3 | 1 | 1 | -3 | 4 | -3 | 0 | 6 | High |
| 34 |  |  | High | 0 | 2 | -1 | -1 | -1 | -1 | -1 | -1 | -1 | -1 | 1 | -1 | 1 | 3 | 1 | 4 | -1 | 3 | -1 | -1 | -1 | -1 | -4 | 0 | -1 | -1 | -1 | -1 | -1 | -1 | -1 | 0 | -1 | 3 | 6 | High |
| 1 |  | 30.496 | Low | -1 | -1 | 1 | 0 | 2 | 1 | -1 | -1 | -1 | -1 | -1 | -1 | 0 | -2 | -2 | 0 | -2 | 0 | -2 | 2 | -2 | 0 | -2 | 0 | 1 | -2 | -2 | -2 | 0 | 1 | -1 | 1 | -1 | -2 | -3 | Low |
| 2 |  | 30.658 | Low | -1 | 1 | -1 | -1 | -1 | -1 | -1 | -1 | -1 | -1 | -1 | -1 | -1 | -1 | -1 | -1 | -1 | 1 | -1 | 1 | -1 | -1 | -1 | -1 | 1 | -1 | -1 | -1 | -1 | 1 | -1 | 1 | -1 | -3 | -3 | Low |
| 3 |  | 31.281 | Low | -4 | -4 | 1 | 1 | -1 | 1 | -1 | -1 | -1 | -1 | -1 | -1 | -2 | 2 | 2 | 2 | -4 | 4 | -4 | 0 | -4 | -4 | -4 | -2 | -1 | -4 | 4 | -2 | 0 | -1 | -1 | 0 | 0 | 0 | 1 | Low |
| 5 |  | 31.554 | Low | -1 | -1 | -1 | -1 | -1 | -1 | -1 | -1 | -1 | -1 | -1 | -1 | -3 | 1 | 1 | 1 | -3 | -1 | -3 | -1 | -1 | -1 | -3 | -1 | 1 | 1 | -3 | -3 | -3 | 1 | -1 | 1 | -1 | -1 | 1 | Low |
| 7 |  | 31.838 | Low | -3 | -3 | -1 | -1 | -1 | -1 | 1 | -1 | -1 | -1 | -1 | -1 | -2 | -1 | -2 | -2 | -4 | 3 | -4 | 1 | -4 | -4 | -4 | -2 | 0 | 0 | -4 | -4 | -2 | 1 | -2 | 1 | -2 | -4 | -2 | Low |
| 8 |  | 31.954 | Low | -2 | -2 | -1 | -1 | -1 | -1 | -1 | -1 | -1 | -1 | -1 | -1 | 0 | 2 | -2 | 0 | -2 | 0 | -2 | 2 | -2 | -2 | -2 | 0 | 0 | -2 | -2 | -2 | 0 | 0 | -1 | 1 | -1 | 0 | -1 | Low |
| 9 |  | 32.081 | Low | -1.5 | -1 | -1 | -1 | -1 | -1 | 2 | 2 | 2 | 2 | -2 | 2 | -1 | -1 | -1 | -1 | 1 | -1 | 1 | -1 | -1 | -1 | -1 | -1 | 1 | -1 | -1 | -1 | -1 | 1 | -1 | 1 | -1 | 1 | 0 | Low |
| 10 |  | 32.747 | Low | -1 | -1 | -1 | -1 | -1 | -1 | -1 | -1 | -1 | -1 | -1 | -1 | -1 | 1 | -1 | 1 | -1 | 1 | -1 | 1 | -1 | -1 | -1 | -1 | 1 | -1 | 1 | -1 | -1 | 1 | -1 | 1 | -1 | -1 | -1 | Low |
| 11 |  | 33.063 | Low | -1 | -1 | -1 | -1 | 1 | -1 | -1 | -1 | -1 | -1 | -1 | -1 | -1 | -1 | -1 | -1 | -1 | -1 | -1 | -1 | -1 | -1 | -2 | -1 | 1 | -1 | -1 | -2 | -0.5 | 1 | -1 | 1 | -1 | 0.5 | -2 | Low |
| 35 |  |  | Low | -2 | -2 | -1 | 1 | -1 | -1 | -1 | -1 | -1 | -1 | -1 | -1 | -3 | -1 | -1 | 1 | 1 | -1 | -1 | 3 | 3 | -1 | -3 | -3 | -3 | -3 | -3 | -3 | -3 | 1 | -1 | -1 | -1 | -7 | -7 | Low |
| 36 |  |  | Low | -1 | -1 | 1 | -1 | -1 | -1 | -1 | -1 | -1 | -1 | 1 | -1 | -1 | -1 | -3 | -1 | -1 | -3 | -1 | -1 | -1 | -3 | -3 | -3 | 1 | -3 | -3 | -3 | -3 | -1 | -1 | 1 | -1 | -3 | -7 | Low |
| 37 |  |  | Low | -2 | -2 | -1 | -1 | -1 | -1 | -1 | -1 | -1 | -1 | -1 | -1 | -1 | -1 | -1 | 1 | -1 | -1 | -1 | -1 | -1 | -1 | -1 | -1 | -1 | -1 | -1 | -1 | -1 | 1 | -1 | -1 | -1 | -1 | -3 | Low |
| 38 |  |  | Low | -2 | -2 | -1 | 1 | -1 | -3 | 1 | -1 | -1 | 1 | -1 | -1 | -2 | -2 | -2 | 0 | -2 | -2 | -2 | -2 | -2 | -2 | -2 | 0 | 0 | -2 | -2 | -2 | 0 | 0 | -1 | 1 | -1 | 0 | -3 | Low |
| 39 |  |  | Low | -4 | -4 | -2 | 0 | -2 | -2 | -1 | -1 | -1 | -1 | -1 | -1 | -2 | 0 | -2 | 1 | 0 | 0 | 0 | 2 | 0 | -2 | -2 | 2 | 2 | 0 | -2 | -2 | 2 | 2 | -2 | 1 | -2 | 0 | -1 | Low |
| 40 |  |  | Low | -2 | -2 | -1 | -1 | -1 | -1 | -1 | -1 | -1 | -1 | -1 | -1 | -1 | 1 | -1 | 1 | 1 | 1 | 1 | 1 | -1 | 1 | -1 | -1 | -1 | -1 | -1 | 1 | 1 | 1 | -1 | 1 | -1 | 3 | -1 | Low |
| 41 |  |  | Low | 0 | 0 | -1 | -1 | -1 | 1 | -1 | 1 | -1 | -1 | 1 | -1 | -1 | 1 | -1 | -1 | -1 | 1 | -1 | 1 | -1 | -1 | -1 | -1 | 1 | -1 | -1 | -1 | -1 | 1 | -1 | 1 | -1 | -1 | 1 | Low |
|  |  |  |  |  |  |  |  |  |  |  |  |  |  |  |  |  |  |  |  |  |  |  |  |  |  |  |  |  |  |  |  |  |  |  |  |  |  |  |  |
|  |  |  |  |  |  |  |  |  |  |  |  |  |  |  |  |  |  |  |  |  |  |  |  |  |  |  |  |  |  |  |  |  |  |  |  |  |  |  |  |
|  |  |  |  |  |  |  |  |  |  |  |  |  |  |  |  |  |  |  |  |  |  |  |  |  |  |  |  |  |  |  |  |  |  |  |  |  |  |  |  |
|  |  |  |  |  |  |  |  |  |  |  |  |  |  |  |  |  |  |  |  |  |  |  |  |  |  |  |  |  |  |  |  |  |  |  |  |  |  |  |  |
|  |  |  |  |  |  |  |  |  |  |  |  |  |  |  |  |  |  |  |  |  |  |  |  |  |  |  |  |  |  |  |  |  |  |  |  |  |  |  |  |
|  |  |  |  |  |  |  |  |  |  |  |  |  |  |  |  |  |  |  |  |  |  |  |  |  |  |  |  |  |  |  |  |  |  |  |  |  |  |  |  |
| 6 | Insufficient Sample | 31.613 | Low | -1 | -1 | -1 | -1 | -1 | -1 | -1 | -1 | -1 | -1 | -1 | -1 | -1 | 1 | -1 | 1 | -1 | -1 | -1 | 1 | -1 | -1 | -2 | -1 | 1 | -1 | -1 | -2 | -0.5 | 1 | -1 | -1 | -1 | 0.5 | -2 | Low |
| 21 | Insufficient Sample | 35.842 | High | -4 | -4 | 1 | 0 | 2 | 2 | 1 | 1 | -1 | 1 | 1 | 1 | 0 | 2 | -4 | 2 | 0 | 2 | 0 | 2 | 0 | 2 | -2 | -4 | -4 | -4 | -4 | -4 | 0 | 0 | -4 | 0 | -4 | 6 | 7 | High |
| 23 | Insufficient Sample | 36.195 | High | -3 | -1 | -1 | -1 | -1 | -1 | -1 | -1 | -1 | -1 | -1 | 1 | -1 | 2 | -1 | 2 | -1 | 1 | -1 | 1 | -1 | 3 | -1 | -1 | 1 | -3 | -3 | -3 | -1 | 1 | -1 | 0 | -2 | 6 | 8 | High |
| 26 | Insufficient Data |  |  |  |  |  |  |  |  |  |  |  |  |  |  |  |  |  |  |  |  |  |  |  |  |  |  |  |  |  |  |  |  |  |  |  |  |  |  |
| 27 | Insufficient Data |  |  |  |  |  |  |  |  |  |  |  |  |  |  |  |  |  |  |  |  |  |  |  |  |  |  |  |  |  |  |  |  |  |  |  |  |  |  |
| 28 | Insufficient Data |  |  |  |  |  |  |  |  |  |  |  |  |  |  |  |  |  |  |  |  |  |  |  |  |  |  |  |  |  |  |  |  |  |  |  |  |  |  |
| 29 | Insufficient Data |  |  |  |  |  |  |  |  |  |  |  |  |  |  |  |  |  |  |  |  |  |  |  |  |  |  |  |  |  |  |  |  |  |  |  |  |  |  |
| 30 | Insufficient Data |  |  |  |  |  |  |  |  |  |  |  |  |  |  |  |  |  |  |  |  |  |  |  |  |  |  |  |  |  |  |  |  |  |  |  |  |  |  |
| 24 | Atypical Deletion |  |  |  |  |  |  |  |  |  |  |  |  |  |  |  |  |  |  |  |  |  |  |  |  |  |  |  |  |  |  |  |  |  |  |  |  |  |  |
| 25 | Atypical Deletion |  |  |  |  |  |  |  |  |  |  |  |  |  |  |  |  |  |  |  |  |  |  |  |  |  |  |  |  |  |  |  |  |  |  |  |  |  |  |
