# Supplementary material for: Candidate modifier genes for immune function in 22q11.2 deletion syndrome
Source: Mol Genet Genomic Med. 2019 Dec 12;8(1):e1057. doi: 10.1002/mgg3.1057 (PMC6978229; doi:10.1002/mgg3.1057)
Supplement: Supplementary file 3 [file MGG3-8-e1057-s003.docx]

| **RANK** | **Gene** | **p-value** | **p-value-ci** | **Score** | **Variants** |  |  |  |  |  |  |  |  |  |
| --- | --- | --- | --- | --- | --- | --- | --- | --- | --- | --- | --- | --- | --- | --- |
| 1 | FASN | 0.00413 | 0.00257,0.00586 | 7.37 | chr17:80046994;1.34;C->T;E->K;0,1 | chr17:80051281;1.34;G->T;L->M;0,1 | chr17:80048790;1.34;C->T;V->I;0,1 | chr17:80044354;1.34;G->A;P->S;0,1 | chr17:80045881;1.34;C->T;E->K;0,1 | chr17:80050420;1.34;C->T;A->T;0,1 | chr17:80050860;1.34;C->T;V->I;0,1 |  |  |  |
| 2 | TBC1D16 | 0.00806 | 0.00496,0.0115 | 6.12 | chr17:77984254;1.31;C->T;E->K;0,1 | chr17:77915983;1.31;A->T;I->N;0,1 | chr17:77921593;5.44;C->T;V->I;0,4 |  |  |  |  |  |  |  |
| 3 | SULT6B1 | 0.00838 | 0.00522,0.0119 | 18.7 | chr2:37414548;5.02;T->C;K->E;0,5 | chr2:37414586;5.02;A->T;V->D;0,5 | chr2:37402356;2.11;C->G;R->S;0,3 | chr2:37415603;6.54;C->T;A->T;0,6 |  |  |  |  |  |  |
| 4 | PCLO | 0.00838 | 0.00522,0.0119 | 7.37 | chr7:82584574;1.34;G->T;Q->K;0,1 | chr7:82764800;1.34;G->A;A->V;0,1 | chr7:82763757;1.34;T->C;T->A;0,1 | chr7:82585758;1.34;G->C;T->S;0,1 | chr7:82785304;1.34;G->A;P->L;0,1 | chr7:82581786;1.34;G->A;T->I;0,1 | chr7:82585022;1.34;T->G;K->N;0,1 |  |  |  |
| 5 | FBN3 | 0.014 | 0.00814,0.0206 | 16.23 | chr19:8137999;1.34;G->A;P->S;0,1 | chr19:8174601;1.34;T->C;N->S;0,1 | chr19:8203113;4.10;C->T;M->I;0,3 | chr19:8168538;1.34;C->T;C->Y;0,1 | chr19:8176947;2.72;T->C;D->G;0,2 | chr19:8201121;4.10;C->T;R->Q;0,3 | chr19:8130939;1.34;C->T;R->Q;0,1 | chr19:8212222;1.34;C->T;R->Q;0,1 | chr19:8145928;1.34;C->T;R->H;0,1 | chr19:8203392;1.34;G->C;L->V;0,1 |
| 6 | OR2L5 | 0.0147 | 0.00867,0.0214 | 6.12 | chr1:248185841;4.08;G->C;V->L;0,3 | chr1:248185883;2.70;G->A;G->S;0,2 | chr1:248185925;1.31;C->T;R->C;0,1 |  |  |  |  |  |  |  |
| 7 | PRR21 | 0.0207 | 0.0135,0.0286 | 7.45 | chr2:240981520;2.71;C->G;V->L;0,2 | chr2:240981418;1.33;T->G;T->P;0,1 | chr2:240982309;1.33;C->T;G->S;0,1 | chr2:240982219;4.08;A->G;S->P;0,3 |  |  |  |  |  |  |
| 8 | BAI2 | 0.0207 | 0.0135,0.0286 | 4.69 | chr1:32198631;1.34;A->G;V->A;0,1 | chr1:32203859;1.34;C->T;R->H;0,1 | chr1:32205602;1.34;C->T;V->M;0,1 | chr1:32193790;1.34;G->A;T->M;0,1 | chr1:32193185;1.34;G->A;R->C;0,1 |  |  |  |  |  |
| 9 | TNK2 | 0.022 | 0.0146,0.0301 | 4.74 | chr3:195594494;4.06;C->T;R->H;0,3 | chr3:195597000;1.32;G->C;L->V;0,1 | chr3:195591056;1.32;C->T;R->H;0,1 |  |  |  |  |  |  |  |
| 10 | ZNF302 | 0.0227 | 0.0151,0.0309 | 4.71 | chr19:35175537;1.34;A->G;K->E;0,1 | chr19:35175306;2.70;G->A;D->N;0,2 | chr19:35176039;1.34;A->G;K->R;0,1 | chr19:35173717;1.34;T->A;H->Q;0,1 |  |  |  |  |  |  |
| 11 | WDR87 | 0.0253 | 0.0174,0.034 | 5.46 | chr19:38376261;1.33;G->C;P->A;0,1 | chr19:38383767;1.33;T->G;Q->P;0,1 | del-chr19-38385614-3;2.11;~->-;~->-;0,3 | chr19:38384689;1.33;C->T;A->T;0,1 | chr19:38384838;1.33;T->A;H->L;0,1 |  |  |  |  |  |
| 12 | TBK1 | 0.0253 | 0.0174,0.034 | 5.02 | chr12:64882317;5.02;T->C;V->A;0,5 |  |  |  |  |  |  |  |  |  |
| 13 | SLC34A3 | 0.028 | 0.0196,0.0371 | 6.05 | chr9:140130522;1.34;G->A;R->H;0,1 | chr9:140128127;1.34;A->C;T->P;0,1 | chr9:140130653;1.34;A->T;I->F;0,1 | chr9:140128387;1.34;C->A;L->M;0,1 | chr9:140127546;2.70;G->C;G->A;0,2 |  |  |  |  |  |
| 14 | ACADL | 0.0286 | 0.0202,0.0378 | 7.45 | chr2:211069376;1.33;G->A;R->W;0,1 | chr2:211085491;5.44;G->A;T->I;0,4 | chr2:211070402;1.33;C->G;G->A;0,1 | chr2:211082773;1.33;T->G;Q->P;0,1 |  |  |  |  |  |  |
| 15 | PAPLN | 0.03 | 0.0175,0.0441 | 7.52 | chr14:73729349;2.71;C->T;A->V;0,2 | chr14:73726151;5.48;A->G;H->R;0,4 | chr14:73729498;1.30;G->A;G->R;0,1 |  |  |  |  |  |  |  |
| 16 | ANKMY1 | 0.03 | 0.0175,0.0441 | 4.69 | chr2:241463461;1.34;T->C;E->G;0,1 | chr2:241463317;1.34;G->A;A->V;0,1 | chr2:241439502;1.34;C->T;M->I;0,1 | chr2:241459832;1.34;C->A;V->L;0,1 | chr2:241492350;1.34;A->G;M->T;0,1 |  |  |  |  |  |
| 17 | ZNF844 | 0.0328 | 0.0197,0.0476 | 4.74 | chr19:12187907;4.06;G->A;V->I;0,3 | chr19:12187151;1.32;C->G;P->A;0,1 | chr19:12186722;1.32;G->A;G->S;0,1 |  |  |  |  |  |  |  |
| 18 | FAT2 | 0.0342 | 0.0208,0.0493 | 12.76 | chr5:150947390;1.34;C->T;R->K;0,1 | chr5:150924726;1.34;C->T;V->M;0,1 | chr5:150921969;2.70;C->T;A->T;0,2 | chr5:150907573;1.34;G->A;A->V;0,1 | chr5:150947664;1.34;C->G;V->L;0,1 | chr5:150924212;1.34;A->G;V->A;0,1 | chr5:150947751;2.70;G->A;P->S;0,2 | chr5:150921921;1.34;C->T;E->K;0,1 | chr5:150885451;1.34;C->T;R->H;0,1 |  |
| 19 | CD101 | 0.0342 | 0.0208,0.0493 | 7.41 | chr1:117556165;1.33;G->A;A->T;0,1 | chr1:117560036;4.06;G->A;R->Q;0,3 | chr1:117559893;1.33;G->T;Q->H;0,1 | chr1:117576697;1.33;G->T;D->Y;0,1 | chr1:117552714;1.33;G->A;V->M;0,1 |  |  |  |  |  |
| 20 | BLOC1S3 | 0.0357 | 0.022,0.051 | 5.02 | chr19:45682876;5.02;C->G;L->V;0,5 |  |  |  |  |  |  |  |  |  |
| 21 | ISPD | 0.0371 | 0.0231,0.0527 | 3.42 | chr7:16445873;1.31;C->T;R->H;0,1 | chr7:16445813;3.42;G->A;A->V;0,3 |  |  |  |  |  |  |  |  |
| 22 | NCOR2 | 0.0385 | 0.0243,0.0544 | 7.39 | chr12:124811972;1.34;T->C;H->R;0,1 | chr12:124848278;1.34;T->G;K->Q;0,1 | chr12:124825196;1.34;C->T;A->T;0,1 | chr12:124819006;1.34;G->A;P->L;0,1 | chr12:124829305;1.34;A->T;S->T;0,1 | chr12:124856830;2.70;C->T;E->K;0,2 |  |  |  |  |
| 23 | TMPRSS6 | 0.0385 | 0.0243,0.0544 | 3.35 | chr22:37462173;1.34;C->T;V->I;0,1 | chr22:37482458;1.34;C->A;V->L;0,1 | chr22:37480861;1.34;G->A;T->M;0,1 | chr22:37471208;1.34;G->A;R->W;0,1 |  |  |  |  |  |  |
| 24 | ALDH1L2 | 0.0399 | 0.0254,0.0561 | 3.35 | chr12:105425570;1.34;C->T;G->E;0,1 | chr12:105425700;1.34;T->C;K->E;0,1 | chr12:105440705;1.34;G->C;L->V;0,1 | chr12:105459085;1.34;T->G;H->P;0,1 |  |  |  |  |  |  |
| 25 | SLC6A5 | 0.0428 | 0.0277,0.0595 | 3.35 | chr11:20676317;1.34;G->A;R->H;0,1 | chr11:20622808;1.34;C->G;P->R;0,1 | chr11:20622937;1.34;C->A;A->E;0,1 | chr11:20676338;1.34;T->C;M->T;0,1 |  |  |  |  |  |  |
| 26 | URGCP | 0.0442 | 0.0289,0.0612 | 3.37 | chr7:43921261;1.33;T->C;T->A;0,1 | chr7:43917441;1.33;C->T;G->S;0,1 | chr7:43916973;2.69;T->C;T->A;0,2 |  |  |  |  |  |  |  |
| 27 | TMEM132C | 0.0471 | 0.0313,0.0645 | 6.05 | chr12:128899748;1.34;G->A;R->Q;0,1 | chr12:128752003;1.34;G->A;S->N;0,1 | chr12:129178435;1.34;C->T;A->V;0,1 | chr12:129190266;1.34;G->A;R->Q;0,1 | chr12:129100757;2.70;C->G;S->R;0,2 |  |  |  |  |  |
| 28 | GRIN3B | 0.0471 | 0.0313,0.0645 | 2.82 | chr19:1003441;1.05;C->T;R->W;0,1 | chr19:1007866;1.05;C->A;P->H;0,1 | chr19:1009413;1.05;G->A;E->K;0,1 | chr19:1008645;0.81;C->A;A->E;1,4 |  |  |  |  |  |  |
| 29 | BVES | 0.0485 | 0.0325,0.0662 | 3.35 | chr6:105581409;1.34;A->G;I->T;0,1 | chr6:105573420;1.34;G->A;R->W;0,1 | chr6:105577342;1.34;C->T;R->Q;0,1 | chr6:105563677;1.34;A->G;L->P;0,1 |  |  |  |  |  |  |
| 30 | PTPRD | 0.0499 | 0.0336,0.0679 | 3.55 | chr9:8497250;3.55;T->C;T->A;0,4 |  |  |  |  |  |  |  |  |  |
| 31 | RGS3 | 0.0499 | 0.0336,0.0679 | 3.42 | chr9:116356430;3.42;G->T;Q->H;0,3 | chr9:116356293;1.31;C->T;R->W;0,1 |  |  |  |  |  |  |  |  |

Supplemental table 2. Complete list of genetic variants for VAAST 2.0 analysis ranked by VAAST score and adjusted p-value using the immune dysregulated (i.e. the high-scoring participants) as the control. This represents modifiers that could be protective against immune dysregulation.
