# Supplementary material for: Candidate modifier genes for immune function in 22q11.2 deletion syndrome
Source: Mol Genet Genomic Med. 2019 Dec 12;8(1):e1057. doi: 10.1002/mgg3.1057 (PMC6978229; doi:10.1002/mgg3.1057)
Supplement: Supplementary file 4 [file MGG3-8-e1057-s004.docx]

| **RANK** | **Gene** | **p-value** | **p-value-ci** | **Score** | **Variants** |  |  |  |  |  |  |  |  |  |  |  |  |  |  |  |  |  |  |  |  |  |  |  |  |  |  |  |  |  |  |  |  |  |  |
| --- | --- | --- | --- | --- | --- | --- | --- | --- | --- | --- | --- | --- | --- | --- | --- | --- | --- | --- | --- | --- | --- | --- | --- | --- | --- | --- | --- | --- | --- | --- | --- | --- | --- | --- | --- | --- | --- | --- | --- |
| 1 | EP300 | 0.00903 | 0.00574,0.0127 | 8.31 | chr22:41574665;1.47;G->A;R->Q;0,1 | chr22:41522003;1.47;A->G;M->V;0,1 | chr22:41562607;1.47;G->A;V->I;0,1 | chr22:41542780;1.47;T->G;S->R;0,1 | chr22:41574940;1.47;A->C;S->R;0,1 | chr22:41574383;2.96;A->C;Q->P;0,2 |  |  |  |  |  |  |  |  |  |  |  |  |  |  |  |  |  |  |  |  |  |  |  |  |  |  |  |  |  |
| 2 | VWA5B1 | 0.00935 | 0.006,0.0131 | 8.38 | chr1:20657463;1.46;G->A;R->Q;0,1 | chr1:20659355;1.46;G->T;V->F;0,1 | chr1:20680275;2.97;C->A;A->G;0,2 | chr1:20645086;4.48;T->A;V->E;0,3 |  |  |  |  |  |  |  |  |  |  |  |  |  |  |  |  |  |  |  |  |  |  |  |  |  |  |  |  |  |  |  |
| 3 | TENM2 | 0.0153 | 0.00919,0.0222 | 8.34 | chr5:167689250;1.47;G->A;S->N;0,1 | chr5:167654983;1.47;A->G;S->G;0,1 | chr5:167489170;4.46;A->G;H->R;0,3 | chr5:167689123;1.47;A->G;I->V;0,1 | chr5:167642133;1.47;C->T;R->C;0,1 |  |  |  |  |  |  |  |  |  |  |  |  |  |  |  |  |  |  |  |  |  |  |  |  |  |  |  |  |  |  |
| 4 | LTBP1 | 0.016 | 0.00972,0.023 | 6.92 | chr2:33172827;1.44;G->A;E->K;0,1 | chr2:33246234;1.44;C->T;P->L;0,1 | chr2:33246146;5.98;G->A;E->K;0,4 |  |  |  |  |  |  |  |  |  |  |  |  |  |  |  |  |  |  |  |  |  |  |  |  |  |  |  |  |  |  |  |  |
| 5 | XKR5 | 0.0168 | 0.0122,0.0216 | 5.37 | chr8:6690239;1.47;C->T;R->Q;0,1 | chr8:6669210;1.47;C->T;G->E;0,1 | chr8:6668953;2.96;G->A;L->F;0,2 | chr8:6679429;1.47;C->A;D->Y;0,1 |  |  |  |  |  |  |  |  |  |  |  |  |  |  |  |  |  |  |  |  |  |  |  |  |  |  |  |  |  |  |  |
| 6 | CNTNAP4 | 0.018 | 0.0113,0.0254 | 5.35 | chr16:76555177;1.47;A->T;I->F;0,1 | chr16:76587325;1.47;C->T;T->M;0,1 | chr16:76311574;1.47;C->T;T->M;0,1 | chr16:76482001;1.47;A->G;K->E;0,1 | chr16:76523711;1.47;A->C;L->F;0,1 |  |  |  |  |  |  |  |  |  |  |  |  |  |  |  |  |  |  |  |  |  |  |  |  |  |  |  |  |  |  |
| 7 | TAF1B | 0.02 | 0.0129,0.0278 | 5.48 | chr2:10074012;1.38;C->A;L->I;0,1 | chr2:10044999;5.48;C->G;D->E;0,4 |  |  |  |  |  |  |  |  |  |  |  |  |  |  |  |  |  |  |  |  |  |  |  |  |  |  |  |  |  |  |  |  |  |
| 8 | SERPINA9 | 0.02 | 0.0129,0.0278 | 5.41 | chr14:94931052;1.46;C->T;V->I;0,1 | chr14:94935807;1.46;G->A;P->L;0,1 | chr14:94936062;4.47;G->A;T->I;0,3 |  |  |  |  |  |  |  |  |  |  |  |  |  |  |  |  |  |  |  |  |  |  |  |  |  |  |  |  |  |  |  |  |
| 9 | CCDC151 | 0.02 | 0.0129,0.0278 | 5.41 | chr19:11545690;1.46;G->A;P->S;0,1 | chr19:11537603;1.46;G->A;T->I;0,1 | chr19:11541539;4.47;G->C;L->V;0,3 |  |  |  |  |  |  |  |  |  |  |  |  |  |  |  |  |  |  |  |  |  |  |  |  |  |  |  |  |  |  |  |  |
| 10 | CCDC114 | 0.024 | 0.0163,0.0325 | 6.87 | chr19:48806094;4.46;C->T;R->H;0,3 | chr19:48800330;1.46;C->T;S->N;0,1 | chr19:48805977;1.46;C->T;R->Q;0,1 | chr19:48800685;1.46;C->T;A->T;0,1 |  |  |  |  |  |  |  |  |  |  |  |  |  |  |  |  |  |  |  |  |  |  |  |  |  |  |  |  |  |  |  |
| 11 | CHTF18 | 0.028 | 0.0196,0.0371 | 6.84 | chr16:839627;1.47;T->C;I->T;0,1 | chr16:840532;1.47;T->C;S->P;0,1 | chr16:845989;1.47;G->A;A->T;0,1 | chr16:842518;2.96;C->T;S->L;0,2 | chr16:845761;1.47;G->A;R->Q;0,1 |  |  |  |  |  |  |  |  |  |  |  |  |  |  |  |  |  |  |  |  |  |  |  |  |  |  |  |  |  |  |
| 12 | COL6A6 | 0.03 | 0.0175,0.0441 | 8.29 | chr3:130346185;1.47;A->G;Y->C;0,1 | chr3:130300513;1.47;G->A;R->H;0,1 | chr3:130293232;1.47;T->C;I->T;0,1 | chr3:130380600;1.47;A->G;I->V;0,1 | chr3:130282383;1.47;C->A;T->K;0,1 | chr3:130282311;1.47;G->A;R->Q;0,1 | chr3:130290007;1.47;T->C;I->T;0,1 |  |  |  |  |  |  |  |  |  |  |  |  |  |  |  |  |  |  |  |  |  |  |  |  |  |  |  |  |
| 13 | COL4A4 | 0.03 | 0.0175,0.0441 | 5.48 | chr2:227915847;5.48;C->T;G->E;0,4 | chr2:228012150;1.38;T->C;K->R;0,1 |  |  |  |  |  |  |  |  |  |  |  |  |  |  |  |  |  |  |  |  |  |  |  |  |  |  |  |  |  |  |  |  |  |
| 14 | E2F1 | 0.0314 | 0.0186,0.0459 | 3.95 | chr20:32264675;2.98;C->T;G->S;0,2 | chr20:32266134;2.98;C->T;G->S;0,2 |  |  |  |  |  |  |  |  |  |  |  |  |  |  |  |  |  |  |  |  |  |  |  |  |  |  |  |  |  |  |  |  |  |
| 15 | OR8G2 | 0.0342 | 0.0208,0.0493 | 4.1 | chr11:124096292;4.10;A->C;N->H;0,4 |  |  |  |  |  |  |  |  |  |  |  |  |  |  |  |  |  |  |  |  |  |  |  |  |  |  |  |  |  |  |  |  |  |  |
| 16 | PINX1 | 0.0342 | 0.0208,0.0493 | 3.95 | chr8:10623254;3.95;C->A;R->I;0,3 | chr8:10623280;1.43;C->G;Q->H;0,1 |  |  |  |  |  |  |  |  |  |  |  |  |  |  |  |  |  |  |  |  |  |  |  |  |  |  |  |  |  |  |  |  |  |
| 17 | BTN2A2 | 0.0342 | 0.0208,0.0493 | 3.88 | chr6:26393009;1.47;G->A;M->I;0,1 | chr6:26392684;1.47;A->G;Y->C;0,1 | chr6:26392629;1.47;G->A;A->T;0,1 | chr6:26390307;1.47;T->G;S->A;0,1 |  |  |  |  |  |  |  |  |  |  |  |  |  |  |  |  |  |  |  |  |  |  |  |  |  |  |  |  |  |  |  |
| 18 | NR3C1 | 0.0353 | 0.0259,0.0455 | 3.95 | chr5:142780337;1.43;C->T;R->K;0,1 | chr5:142779317;3.95;T->C;N->S;0,3 |  |  |  |  |  |  |  |  |  |  |  |  |  |  |  |  |  |  |  |  |  |  |  |  |  |  |  |  |  |  |  |  |  |
| 19 | RRBP1 | 0.0357 | 0.022,0.051 | 5.41 | chr20:17595399;1.46;C->T;E->K;0,1 | chr20:17617260;1.46;G->A;R->W;0,1 | chr20:17602571;4.47;A->G;C->R;0,3 |  |  |  |  |  |  |  |  |  |  |  |  |  |  |  |  |  |  |  |  |  |  |  |  |  |  |  |  |  |  |  |  |
| 20 | SPON2 | 0.0357 | 0.022,0.051 | 4.1 | chr4:1165131;4.10;C->T;A->T;0,4 |  |  |  |  |  |  |  |  |  |  |  |  |  |  |  |  |  |  |  |  |  |  |  |  |  |  |  |  |  |  |  |  |  |  |
| 21 | CYP2A7 | 0.0357 | 0.022,0.051 | 3.9 | chr19:41383115;1.46;G->A;R->W;0,1 | chr19:41383849;1.46;C->G;S->T;0,1 | chr19:41383141;2.96;C->T;R->H;0,2 |  |  |  |  |  |  |  |  |  |  |  |  |  |  |  |  |  |  |  |  |  |  |  |  |  |  |  |  |  |  |  |  |
| 22 | GPC1 | 0.0357 | 0.022,0.051 | 3.88 | chr2:241401740;1.47;G->A;R->H;0,1 | chr2:241404336;1.47;C->T;R->C;0,1 | chr2:241404098;1.47;G->A;V->M;0,1 | chr2:241401673;1.47;G->A;G->R;0,1 |  |  |  |  |  |  |  |  |  |  |  |  |  |  |  |  |  |  |  |  |  |  |  |  |  |  |  |  |  |  |  |
| 23 | PIEZO1 | 0.0357 | 0.022,0.051 | 3.88 | chr16:88782483;1.47;C->T;E->K;0,1 | chr16:88789687;1.47;C->T;R->Q;0,1 | chr16:88800804;1.47;C->T;V->M;0,1 | chr16:88782050;1.47;G->A;P->L;0,1 |  |  |  |  |  |  |  |  |  |  |  |  |  |  |  |  |  |  |  |  |  |  |  |  |  |  |  |  |  |  |  |
| 24 | MPND | 0.0371 | 0.0231,0.0527 | 3.95 | chr19:4359191;3.95;C->T;P->L;0,3 | chr19:4354403;1.43;G->C;V->L;0,1 |  |  |  |  |  |  |  |  |  |  |  |  |  |  |  |  |  |  |  |  |  |  |  |  |  |  |  |  |  |  |  |  |  |
| 25 | COBL | 0.0371 | 0.0231,0.0527 | 3.9 | chr7:51152902;1.46;G->C;R->G;0,1 | chr7:51097255;2.96;G->A;S->F;0,2 | chr7:51152904;1.46;T->C;N->S;0,1 |  |  |  |  |  |  |  |  |  |  |  |  |  |  |  |  |  |  |  |  |  |  |  |  |  |  |  |  |  |  |  |  |
| 26 | TTN | 0.0385 | 0.0243,0.0544 | 41.92 | chr2:179431963;1.47;A->T;V->D;0,1 | chr2:179642515;1.47;A->G;F->L;0,1 | chr2:179643775;1.47;C->T;G->D;0,1 | chr2:179441295;1.47;T->C;S->G;0,1 | chr2:179440163;1.47;C->G;G->R;0,1 | chr2:179598170;1.47;C->T;V->M;0,1 | chr2:179585312;1.47;G->A;S->L;0,1 | chr2:179456220;1.47;C->T;V->M;0,1 | chr2:179453429;1.47;G->A;T->I;0,1 | chr2:179404550;1.47;G->A;R->C;0,1 | chr2:179479607;1.47;G->A;P->S;0,1 | chr2:179430596;1.47;A->G;F->L;0,1 | chr2:179418418;1.47;C->T;E->K;0,1 | chr2:179486223;1.47;C->T;D->N;0,1 | chr2:179453426;1.47;C->T;R->Q;0,1 | chr2:179410693;1.47;A->G;I->T;0,1 | chr2:179600475;1.47;C->T;A->T;0,1 | chr2:179596181;1.47;G->C;T->S;0,1 | chr2:179586604;2.97;C->G;D->H;0,2 | chr2:179567340;1.47;G->A;H->Y;0,1 | chr2:179462331;1.47;C->G;V->L;0,1 | chr2:179430193;1.47;T->C;Y->C;0,1 | chr2:179418346;1.47;C->T;V->M;0,1 | chr2:179600511;1.47;G->C;P->A;0,1 | chr2:179482089;1.47;C->T;R->H;0,1 | chr2:179634961;2.97;C->A;V->F;0,2 | chr2:179393859;1.47;A->G;I->T;0,1 | chr2:179399539;1.47;T->C;I->V;0,1 | chr2:179416989;1.47;A->G;I->T;0,1 | chr2:179393691;1.47;G->A;T->I;0,1 | chr2:179414976;1.47;G->A;P->L;0,1 | chr2:179419792;2.97;G->A;S->F;0,2 | chr2:179536955;1.47;C->T;R->H;0,1 | chr2:179632598;1.47;C->T;R->Q;0,1 | chr2:179449131;1.47;G->A;S->L;0,1 |
| 27 | OR6A2 | 0.0386 | 0.0288,0.0492 | 4.1 | chr11:6816792;4.10;T->C;I->V;0,4 |  |  |  |  |  |  |  |  |  |  |  |  |  |  |  |  |  |  |  |  |  |  |  |  |  |  |  |  |  |  |  |  |  |  |
| 28 | OR4D9 | 0.0399 | 0.0254,0.0561 | 4.1 | chr11:59282861;4.10;A->G;Q->R;0,4 |  |  |  |  |  |  |  |  |  |  |  |  |  |  |  |  |  |  |  |  |  |  |  |  |  |  |  |  |  |  |  |  |  |  |
| 29 | ZSCAN5B | 0.0399 | 0.0254,0.0561 | 3.88 | chr19:56703271;1.47;G->A;A->V;0,1 | chr19:56704261;1.47;T->C;E->G;0,1 | chr19:56702286;1.47;G->T;T->N;0,1 | chr19:56704169;1.47;C->T;D->N;0,1 |  |  |  |  |  |  |  |  |  |  |  |  |  |  |  |  |  |  |  |  |  |  |  |  |  |  |  |  |  |  |  |
| 30 | FMO3 | 0.0413 | 0.0311,0.0522 | 3.9 | chr1:171076952;1.46;C->T;P->L;0,1 | chr1:171077274;2.96;G->T;G->V;0,2 | chr1:171077292;1.46;G->A;R->H;0,1 |  |  |  |  |  |  |  |  |  |  |  |  |  |  |  |  |  |  |  |  |  |  |  |  |  |  |  |  |  |  |  |  |
| 31 | DCAF4 | 0.0414 | 0.0266,0.0578 | 3.9 | chr14:73421186;2.96;C->T;L->F;0,2 | chr14:73425364;1.46;C->T;R->C;0,1 | chr14:73421159;1.46;A->G;S->G;0,1 |  |  |  |  |  |  |  |  |  |  |  |  |  |  |  |  |  |  |  |  |  |  |  |  |  |  |  |  |  |  |  |  |
| 32 | ACTN1 | 0.0414 | 0.0266,0.0578 | 3.9 | chr14:69341653;1.46;T->A;T->S;0,1 | chr14:69341658;2.96;G->A;P->L;0,2 | chr14:69341670;1.46;G->A;A->V;0,1 |  |  |  |  |  |  |  |  |  |  |  |  |  |  |  |  |  |  |  |  |  |  |  |  |  |  |  |  |  |  |  |  |
| 33 | CASP5 | 0.0414 | 0.0266,0.0578 | 3.88 | chr11:104878012;1.47;T->G;K->N;0,1 | chr11:104872901;1.47;G->A;R->C;0,1 | chr11:104879658;1.47;T->A;K->N;0,1 | chr11:104869614;1.47;G->A;P->L;0,1 |  |  |  |  |  |  |  |  |  |  |  |  |  |  |  |  |  |  |  |  |  |  |  |  |  |  |  |  |  |  |  |
| 34 | KIAA1009 | 0.0428 | 0.0277,0.0595 | 3.9 | chr6:84881373;1.46;C->T;R->Q;0,1 | chr6:84904604;2.96;G->C;S->C;0,2 | chr6:84879058;1.46;G->A;R->W;0,1 |  |  |  |  |  |  |  |  |  |  |  |  |  |  |  |  |  |  |  |  |  |  |  |  |  |  |  |  |  |  |  |  |
| 35 | MAML2 | 0.0428 | 0.0277,0.0595 | 3.9 | chr11:96074927;1.46;C->T;A->T;0,1 | chr11:95713077;1.46;C->T;V->I;0,1 | chr11:95826485;2.96;T->C;Q->R;0,2 |  |  |  |  |  |  |  |  |  |  |  |  |  |  |  |  |  |  |  |  |  |  |  |  |  |  |  |  |  |  |  |  |
| 36 | NOL8 | 0.0428 | 0.0277,0.0595 | 3.88 | chr9:95078413;1.47;T->C;K->R;0,1 | chr9:95080949;1.47;A->G;F->S;0,1 | chr9:95072500;1.47;C->T;R->H;0,1 | chr9:95077691;1.47;A->G;S->P;0,1 |  |  |  |  |  |  |  |  |  |  |  |  |  |  |  |  |  |  |  |  |  |  |  |  |  |  |  |  |  |  |  |
| 37 | ANXA9 | 0.0428 | 0.0277,0.0595 | 2.79 | chr1:150955582;2.79;A->G;M->V;1,6 |  |  |  |  |  |  |  |  |  |  |  |  |  |  |  |  |  |  |  |  |  |  |  |  |  |  |  |  |  |  |  |  |  |  |
| 38 | RPL8 | 0.0456 | 0.0301,0.0629 | 4.1 | chr8:146016869;4.10;T->C;I->V;0,4 |  |  |  |  |  |  |  |  |  |  |  |  |  |  |  |  |  |  |  |  |  |  |  |  |  |  |  |  |  |  |  |  |  |  |
| 39 | CRTAC1 | 0.0456 | 0.0301,0.0629 | 3.88 | chr10:99683022;1.47;T->C;K->R;0,1 | chr10:99683122;1.47;G->A;R->C;0,1 | chr10:99664433;1.47;C->T;R->H;0,1 | chr10:99640120;1.47;C->T;V->M;0,1 |  |  |  |  |  |  |  |  |  |  |  |  |  |  |  |  |  |  |  |  |  |  |  |  |  |  |  |  |  |  |  |
| 40 | CAPRIN2 | 0.0456 | 0.0301,0.0629 | 3.88 | chr12:30884336;1.47;A->G;M->T;0,1 | chr12:30877343;1.47;T->C;T->A;0,1 | chr12:30867934;1.47;C->T;R->K;0,1 | chr12:30878976;1.47;G->C;P->R;0,1 |  |  |  |  |  |  |  |  |  |  |  |  |  |  |  |  |  |  |  |  |  |  |  |  |  |  |  |  |  |  |  |
| 41 | SEC16A | 0.0471 | 0.0313,0.0645 | 6.84 | chr9:139369079;1.47;T->C;T->A;0,1 | chr9:139360719;1.47;G->T;H->Q;0,1 | chr9:139345749;1.47;G->A;P->S;0,1 | chr9:139366451;1.47;T->C;H->R;0,1 | chr9:139348749;2.96;G->A;A->V;0,2 |  |  |  |  |  |  |  |  |  |  |  |  |  |  |  |  |  |  |  |  |  |  |  |  |  |  |  |  |  |  |
| 42 | OR1E1 | 0.0471 | 0.0313,0.0645 | 5.48 | chr17:3301278;5.48;C->T;A->T;0,4 | chr17:3300824;1.38;T->A;N->I;0,1 |  |  |  |  |  |  |  |  |  |  |  |  |  |  |  |  |  |  |  |  |  |  |  |  |  |  |  |  |  |  |  |  |  |
| 43 | VARS2 | 0.0471 | 0.0313,0.0645 | 3.9 | chr6:30890535;2.96;T->C;I->T;0,2 | chr6:30893700;1.46;G->A;R->Q;0,1 | chr6:30883607;1.46;G->A;A->T;0,1 |  |  |  |  |  |  |  |  |  |  |  |  |  |  |  |  |  |  |  |  |  |  |  |  |  |  |  |  |  |  |  |  |
| 44 | SKIDA1 | 0.0471 | 0.0313,0.0645 | 3.9 | chr10:21806333;2.96;C->A;R->L;0,2 | chr10:21805454;1.46;G->A;A->V;0,1 | chr10:21806350;1.46;G->C;D->E;0,1 |  |  |  |  |  |  |  |  |  |  |  |  |  |  |  |  |  |  |  |  |  |  |  |  |  |  |  |  |  |  |  |  |
| 45 | LUZP1 | 0.0485 | 0.0325,0.0662 | 5.48 | chr1:23419283;2.96;G->A;T->I;0,2 | chr1:23418803;4.51;G->C;S->C;0,3 |  |  |  |  |  |  |  |  |  |  |  |  |  |  |  |  |  |  |  |  |  |  |  |  |  |  |  |  |  |  |  |  |  |
| 46 | SPTY2D1 | 0.0485 | 0.0325,0.0662 | 4.1 | chr11:18636481;4.10;C->T;R->Q;0,4 |  |  |  |  |  |  |  |  |  |  |  |  |  |  |  |  |  |  |  |  |  |  |  |  |  |  |  |  |  |  |  |  |  |  |
| 47 | PRR14L | 0.0485 | 0.0325,0.0662 | 4.1 | chr22:32108475;4.10;C->T;V->I;0,4 |  |  |  |  |  |  |  |  |  |  |  |  |  |  |  |  |  |  |  |  |  |  |  |  |  |  |  |  |  |  |  |  |  |  |
| 48 | SNCAIP | 0.0485 | 0.0325,0.0662 | 3.88 | chr5:121785560;1.47;C->T;T->M;0,1 | chr5:121786667;1.47;G->C;E->Q;0,1 | chr5:121759069;1.47;G->A;V->M;0,1 | chr5:121759097;1.47;C->T;S->L;0,1 |  |  |  |  |  |  |  |  |  |  |  |  |  |  |  |  |  |  |  |  |  |  |  |  |  |  |  |  |  |  |  |
| 49 | PLXNA2 | 0.0499 | 0.0336,0.0679 | 3.95 | chr1:208201360;1.43;A->T;S->R;0,1 | chr1:208212224;3.95;C->T;V->M;0,3 |  |  |  |  |  |  |  |  |  |  |  |  |  |  |  |  |  |  |  |  |  |  |  |  |  |  |  |  |  |  |  |  |  |
| 50 | ELN | 0.0499 | 0.0336,0.0679 | 3.88 | chr7:73459575;1.47;G->T;V->L;0,1 | chr7:73482987;1.47;G->A;G->D;0,1 | chr7:73472000;1.47;A->G;K->R;0,1 | chr7:73456952;1.47;G->A;A->T;0,1 |  |  |  |  |  |  |  |  |  |  |  |  |  |  |  |  |  |  |  |  |  |  |  |  |  |  |  |  |  |  |  |

Supplemental table 3. Complete list of genetic variants for VAAST 2.0 analysis ranked by VAAST score and adjusted p-value using the immune-competent group (i.e. the low-scoring participants) as the control group. This list represents modifiers that could be associated with increased risk of immune dysregulation.
